# Natural Convection Flow near a Vertical Plate that Applies a Shear Stress to a Viscous Fluid

**DOI:** 10.1371/journal.pone.0078352

**Published:** 2013-11-22

**Authors:** Qammar Rubbab, Dumitru Vieru, Corina Fetecau, Constantin Fetecau

**Affiliations:** 1 Department of Mathematics, Air University, Multan Campus, Pakistan; 2 Department of Theoretical Mechanics, Technical University of Iasi, Iasi, Romania; 3 Department of Mathematics, Technical University of Iasi, Iasi, Romania; 4 Member of Academy of Romanian Scientists, Bucuresti, Romania; Vrije Universiteit, Netherlands

## Abstract

The unsteady natural convection flow of an incompressible viscous fluid near a vertical plate that applies an arbitrary shear stress to the fluid is studied using the Laplace transform technique. The fluid flow is due to both the shear and the heating of the plate. Closed-form expressions for velocity and temperature are established under the usual Boussinesq approximation. For illustration purposes, two special cases are considered and the influence of pertinent parameters on the fluid motion is graphically underlined. The required time to reach the steady state in the case of oscillating shear stresses on the boundary is also determined.

## Introduction

Free convection flow past a vertical plate has been extensively studied and continues to receive much attention due to its industrial and technological applications. It is encountered, for instance, in the cooling of nuclear reactors or in the study of environmental heat transfer processes. Soundalgekar and Gupta [1] and Singh and Kumar [2] studied the free convection flow over an accelerated, respectively exponentially accelerated, infinite vertical plate, while Merkin [3] presented a discussion on the similarity solutions. Transient free convection flow past an infinite vertical plate has been studied, for instance, by Ingham [4] and Das *et al.* [5]. Many other unsteady free convection flows over an infinite plate, taking into account radiative, porous or magnetic effects, have also been investigated under different sets of boundary conditions. Some of the most recent and interesting results seem to be those obtained by Toki and Tokis [6], Toki [7], Rajesh [8], Narahari and Ishak [9], Narahari and Nayan [10], Samiulhaq *et al.* [11], [12] and Narahari and Debnath [13]. In their work, Narahari and Debnath, for instance, consider the unsteady magnetohydrodynamic free convection flow with constant heat flux and heat generation or absorption, and obtain exact solutions when the plate is exponentially or uniformly accelerated. Free convective boundary layer flows with Newtonian heating or thermal slip conditions have been numerically investigated by Uddin *et al.* [14], respectively Khan *et al.* [15]. However, in all these works, boundary conditions for velocity are imposed, although in some problems, the force applied on the boundary is specified. In this case, unlike the usual “no slip” condition, a boundary condition on the shear stress has to be used.

It is also important to specify that the “no slip” boundary condition may not be necessarily applicable to flows of some polymeric fluids that can slip or slide on the boundary. Thus, the shear stress boundary condition is particularly meaningful (see also Renardy [16]) and the first exact solutions for fluid motions with shear-stress on the boundary seem to be those of Waters and King [17] and Bandelli *et al.* [18]. Over time, many other papers studied motion problems in which the shear stress is given on the boundary, but they did not take into consideration thermal effects. In a recent paper [19], the unsteady free convection flow near a vertical plate that applies an arbitrary shear stress to the fluid is studied. Porous and radiative effects are taken into consideration, but the plate is kept at a constant temperature.

In the present study, closed-form solutions corresponding to the unsteady free convection flow of an incompressible viscous fluid near an infinite vertical plate with exponential heating are derived using Laplace transforms. The thermal boundary condition is chosen so that the temperature variations in the flow field would be sufficiently large or small and some possible situations would appear as limiting cases. The plate is initially at rest and then suddenly it applies an arbitrary shear stress to the fluid. The convective effects and viscous dissipation are neglected, and exact solutions for dimensionless temperature, Nusselt number and velocity are presented in simple forms. Such solutions are uncommon in the literature. They can generate a wide range of exact solutions for different motions with technical relevance. In order to illustrate their importance, two special cases are considered, and the effects of pertinent parameters on the temperature and velocity distributions are graphically underlined. The required time to reach the steady state for oscillating shears on the boundary is also determined.

## Statement of the Problem

Let us consider the unsteady free convection flow of an incompressible viscous fluid near an infinite vertical plate with exponential heating. The *x*-axis is taken along the plate in the vertical direction and the *y*-axis is normal to the plate. Initially, the plate and the fluid are at rest at the constant temperature 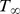. After 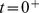 the plate, whose temperature is raised or lowered to 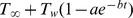, applies a shear stress 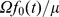 to the fluid where 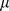 is the coefficient of viscosity and 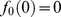. The fluid is gradually moved and its flow is considered to be laminar without any pressure gradient in the flow direction. Assuming that the convective effects and viscous dissipation are negligible and bearing in mind the boundary layer and Boussinesq approximations, the governing equations for such a flow are [20]–[22]

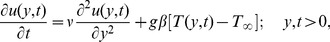
(1)

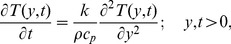
(2)where 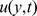 is the fluid velocity along the *x*-axis, 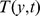 the temperature, 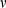 the kinematic viscosity, *g* the acceleration due to the gravity, 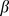 the volumetric coefficient of thermal expansion, *k* the thermal conductivity, 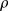 the density and 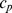 is the specific heat of the fluid at constant pressure.

The appropriate initial and boundary conditions are
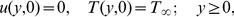
(3)

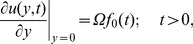
(4)

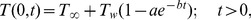
(5)

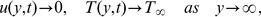
(6)where 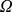, 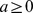 and 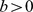 are constants. The dimensions of 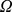 and b are 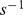.

Introducing the next non-dimensional variables and functions
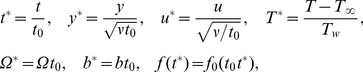
(7)where 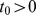 is a characteristic time and dropping the star notation, we obtain the next dimensionless initial and boundary-value problem
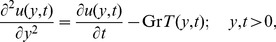
(8)

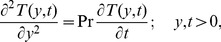
(9)

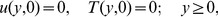
(10)

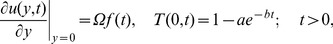
(11)

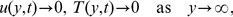
(12)where 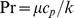 is the Prandtl number and 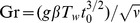 is the Grashof number. The function 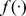 is assumed to be piecewise continuous and of exponential order for 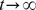 [23].

## Method of Solution and Analytic Results

In order to determine the solution of the initial and boundary-value problem (8)–(12) we use the Laplace transform technique to eliminate the variable *t*. Exact analytical expressions for dimensionless temperature and velocity fields will be separately established for 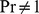 and 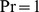. Nusselt number is also determined.

### The case 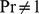


Applying the Laplace transform to (9) and using the corresponding initial and boundary conditions for temperature, we find the next problem in the transform domain
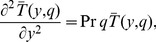
(13)

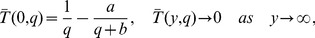
(14)where 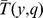 is the Laplace transform of 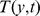 and *q* is the transform parameter.

The solution of the problem (13) and (14) is given by
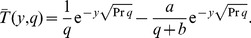
(15)


Now, applying the inverse Laplace transform to this last equality, we find for the dimensionless temperature 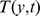 the expression (see also [24])
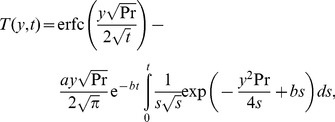
(16)which clearly satisfies the initial condition (10)_2_ and the natural condition (12)_2_ at infinity. However, in this form, the boundary condition (11)_2_ seems not to be satisfied. In order to eliminate this drawback, we present for 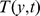 the equivalent but elegant form (see also [25])
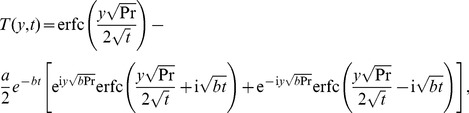
(17)in terms of the complementary error function 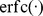.

The Nusselt number Nu, which measures the heat transfer rate at the plate, can be obtained using any of the above expressions for temperature. However, we must be very careful because the immediate results can be misleading. If we use (16), for instance, the correct result
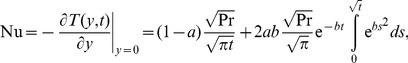
(18)is obtained taking into account the fact that
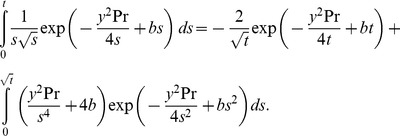



For velocity, we apply the Laplace transform to (8) and use both the corresponding initial and boundary conditions and (15) for 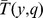. The problem in the transform domain is given by
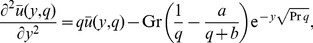
(19)

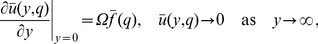
(20)where 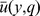 and 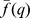 are the Laplace transforms of 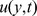 and 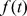, respectively. The solution of the boundary value problem (19) and (20) is given by
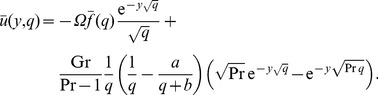
(21)


Inverting this result, we get for 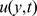 the simple form
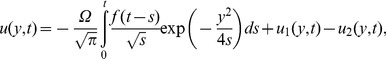
(22)where
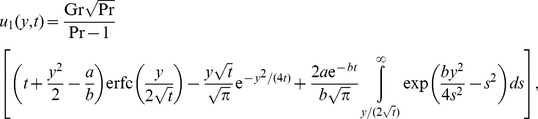
(23)

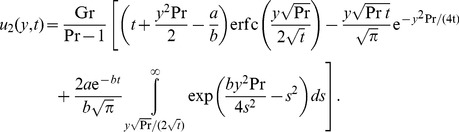
(24)It is worth pointing out that the two integrals from (23) and (24) can be further processed (see also [6]) in order to provide for 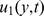 and 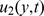 the explicit and elegant forms
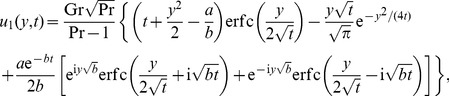
(25)

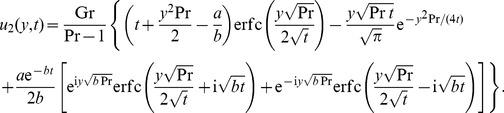
(26)


Direct computations show that 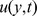, given by (22), satisfies all imposed initial and boundary conditions. In order to prove the boundary condition (11)_1_, for instance, it is sufficient to show that the derivative with respect to *y* of the first term from (22) can be written in the equivalent form
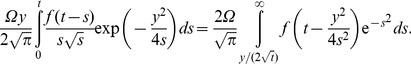
(27)


### The case 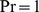


The temperature distribution corresponding to this case can be directly obtained by making 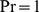 in any of equations (16) or (17). The equality (17), for instance, becomes
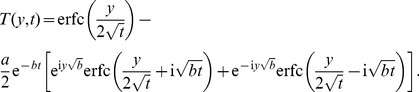
(28)


For velocity, by making 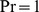 into (19) and using the same method as before, we find that
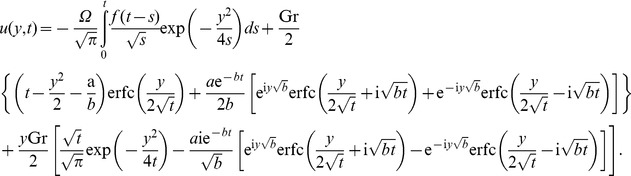
Of course, if the plate is maintained at a constant temperature, namely 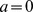, the above solutions are substantially simplified. In the case 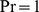, for example, they take the simple forms (see [22] for temperature and Nusselt number)
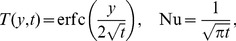
(30)

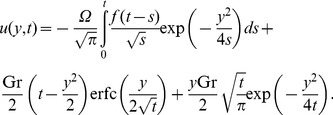
(31)


In the absence of thermal effects, as expected, in all cases the velocity is reduced to its mechanical component
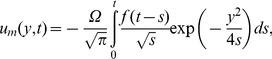
(32)which is equivalent to the similar result obtained from [19]. Consequently, the fluid velocity 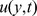 can be written as a sum of its mechanical and thermal components 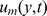, respectively, 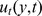.

## Applications

The previous solutions can be used to obtain the dimensionless temperature, Nusselt number and velocity distributions corresponding to any motion problem with physical relevance. For illustration purposes, two special cases are considered, and the corresponding solutions are graphically discussed.

### Flow induced by a plate that applies a constant shear to the fluid

Suppose that the vertical plate suddenly applies a constant shear stress 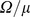 to the fluid. In this case the function 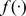 is identical to the Heaviside unit step function 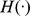 and
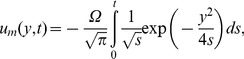
(33)can be written in the simple but equivalent form
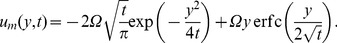
(34)The temperature distribution is given by any of equations (16), (17) or (28), while the corresponding velocity is obtained introducing (34) into (22) or (29).

### Flow due to a plate that applies oscillating shears to the fluid

Let us now assume that the vertical plate applies an oscillatory shear 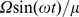 or 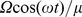 to the fluid. The function 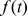 corresponding to these motions is 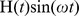, respectively 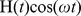 and the associated mechanical components of velocity are
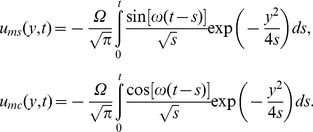
(35)


The temperature distribution is the same as before, but the corresponding velocities are obtained by introducing (35) in (22) or (29). The starting solutions (35) are important for those who want to eliminate the transients from their experiments. They can be written as a sum of steady-state and transient solutions. In order to determine the required time to reach the steady-state, namely the time after which the fluid flows according to the steady-state solutions, we present here the steady-state solutions only, namely (see also [19])
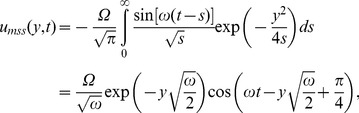
(36)

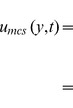
(37)


The steady-state components (36) and (37), unlike the starting solutions (35), differ by a phase shift. This is the reason why we presented the solutions separately, corresponding to the sine or cosine shear stresses on the boundary.

## Numerical Results and Discussion

The unsteady natural convection flow of an incompressible viscous fluid near an infinite vertical plate with exponential heating is analytically studied. Initially, the plate is at rest and then it suddenly applies an arbitrary time-dependent shear stress to the fluid. Exact solutions are established for dimensionless temperature, Nusselt number and velocity without any restriction. Equivalent forms are presented for the temperature, while the fluid velocity 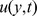 is presented as a sum of mechanical 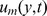 and thermal 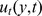 components. In order to illustrate the theoretical and practical value of general solutions, two special cases corresponding to constant or oscillating shears on the boundary are considered. Such solutions, in addition to serving as approximations to some specific initial-boundary value problems, can also be used as tests to verify numerical schemes that are developed to study more complex unsteady flow problems.

**Figure 1:**
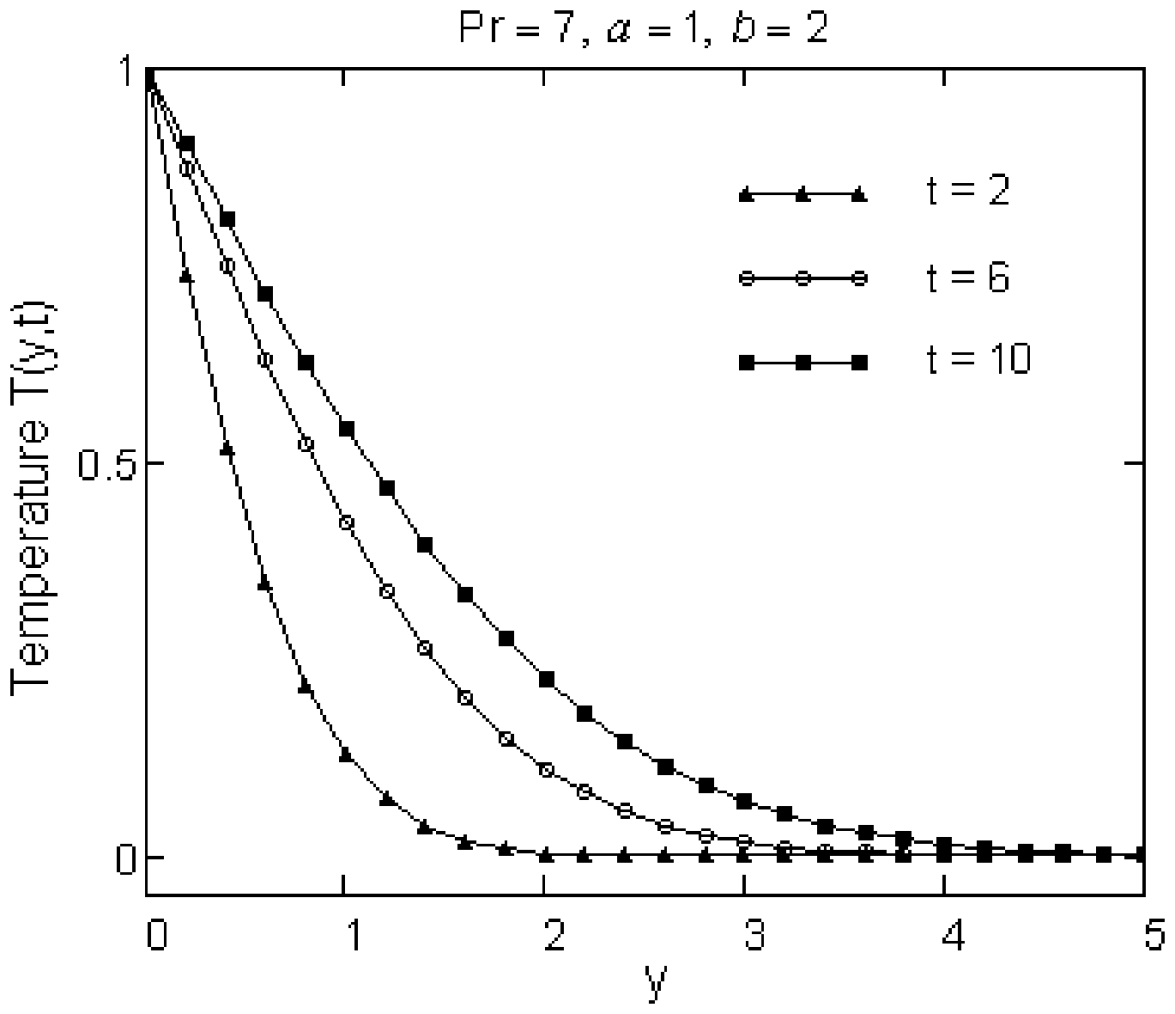
Temperature profiles for different values of *t*.

**Figure 2:**
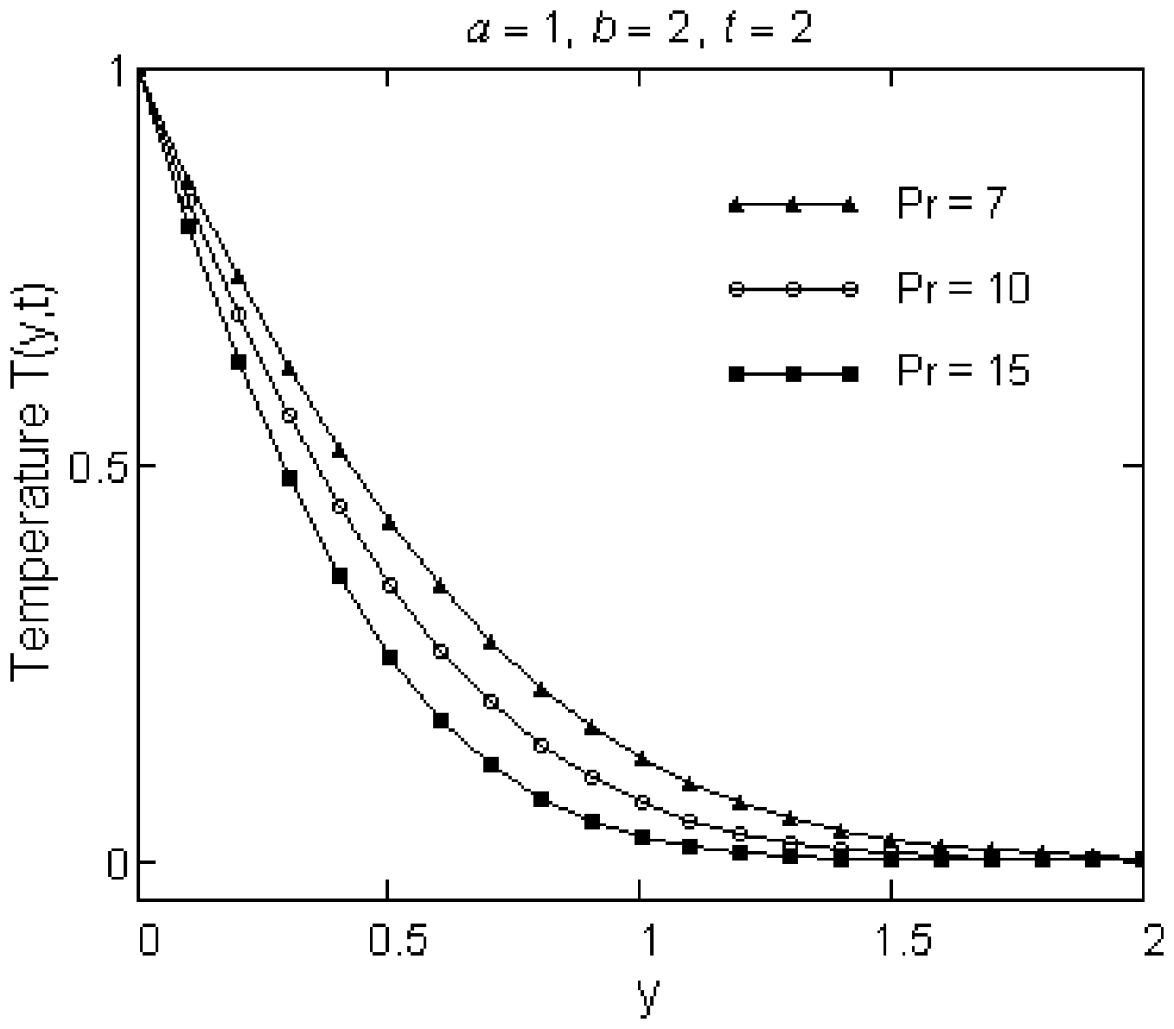
Temperature profiles for different values of Pr.

**Figure 3:**
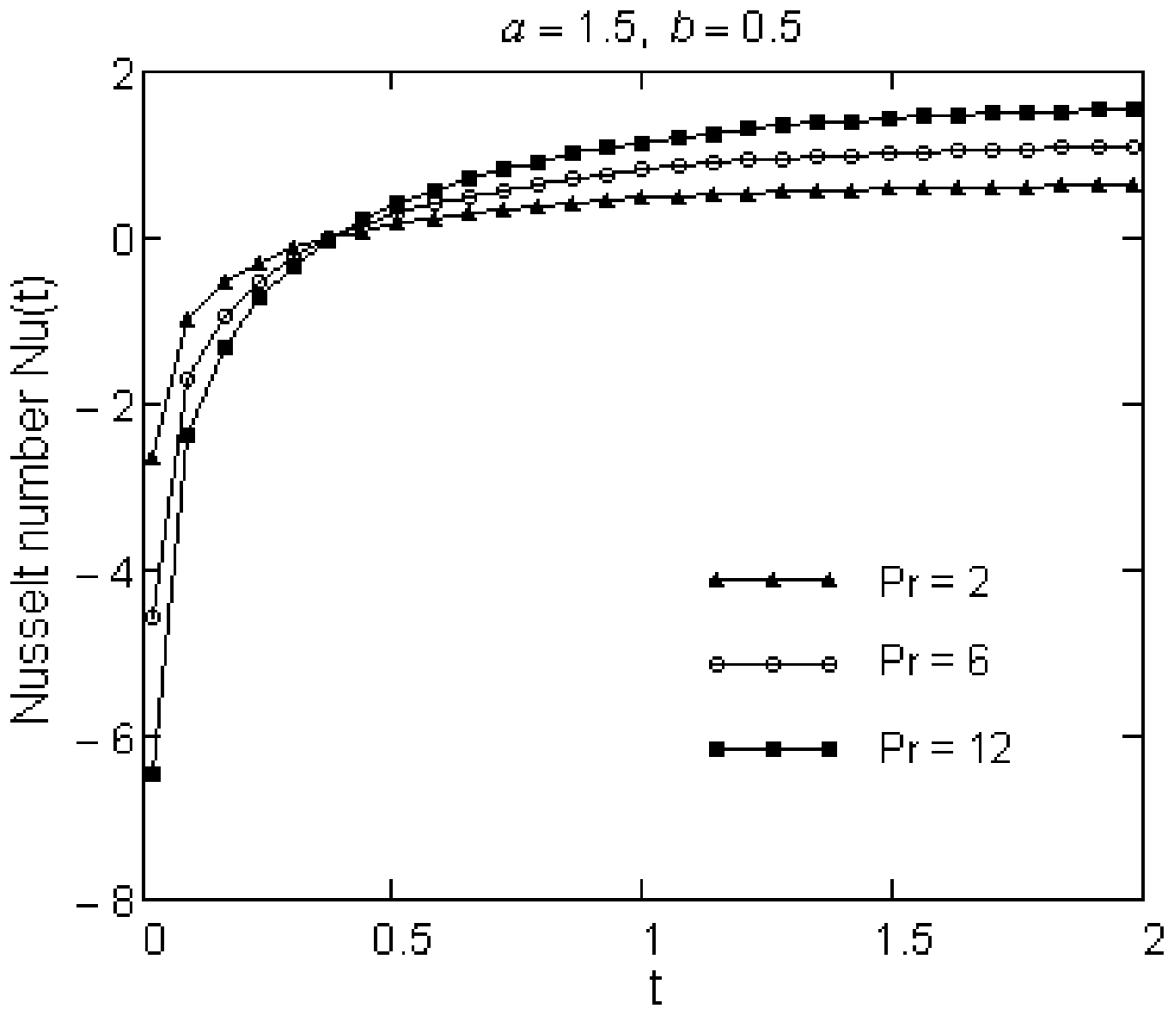
Variation of Nusselt number for different values of Pr.

In order to get some physical insight of the results corresponding to a constant shear on the boundary, some numerical calculations have been carried out for different values of pertinent parameters that describe the flow characteristics. Fig. 1 presents the temperature profiles against y at different times and fixed values of the constants 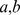 and Prandtl number Pr. The fluid temperature is an increasing function with respect to time and tends to a steady-state as the time increases. It is also found that the thermal boundary layer thickens over time. The influence of Prandtl number Pr on the temperature is shown in Fig. 2 for 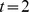. It is clearly seen that an increase of Pr implies a significant decrease of the temperature. The temperature of the fluid, as before, smoothly decreases from a maximum value at the boundary to a minimum value for large values of *y*. Its values at any distance *y* are always higher for 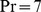 than for 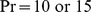. Furthermore, an increase of Prandtl number implies a decreasing of the thermal boundary layer thickness. This is possible because greater values of Pr are equivalent to decreasing thermal conductivity. The variation of Nusselt number over time is shown in Fig. 3 for three values of Pr. Up to a critical value of time 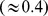, the Nusselt number profiles decrease with respect to Pr and then increase. Furthermore, as time advances, the Nusselt number increases and becomes constant in time.

**Figure 4:**
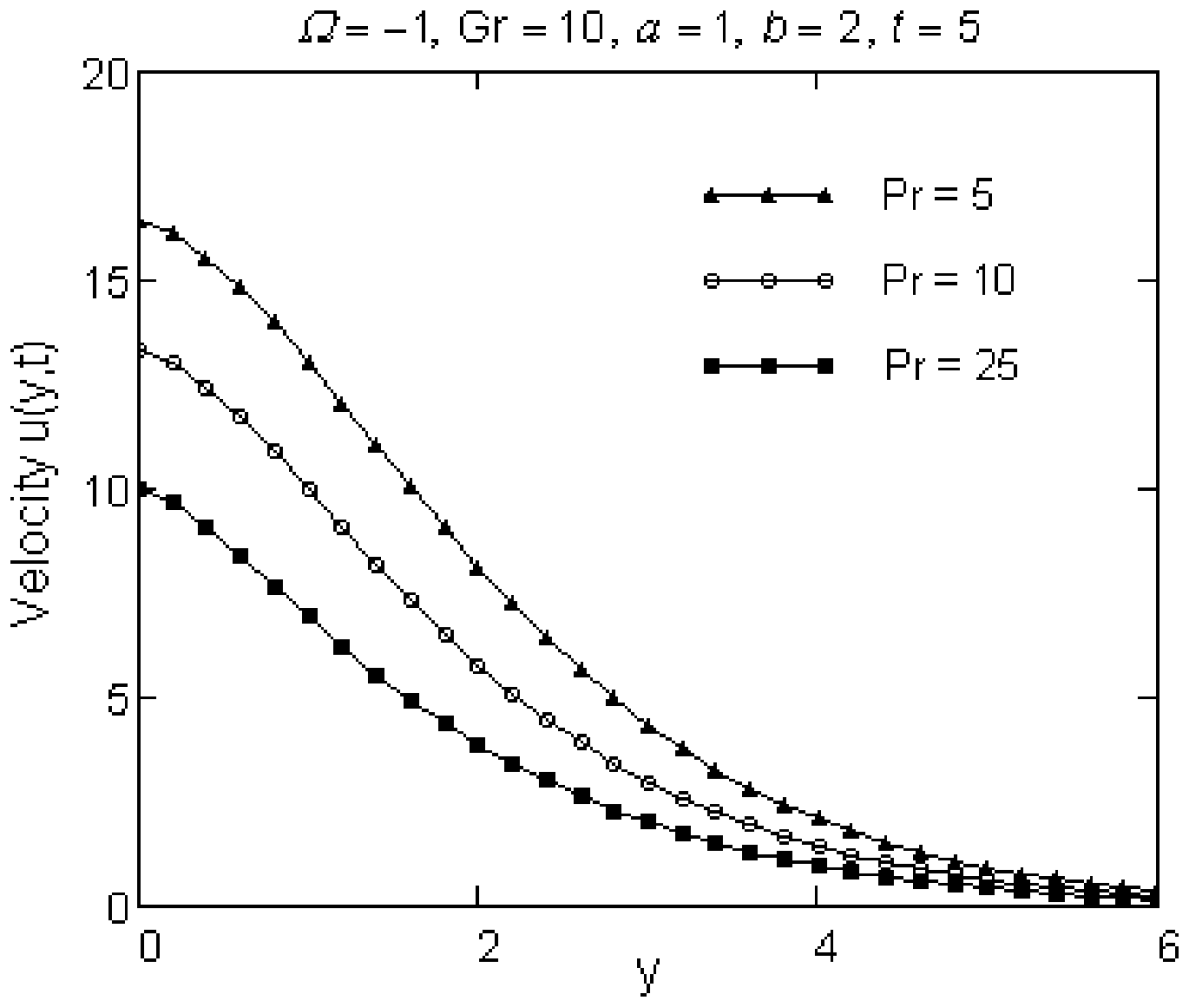
Velocity profiles for different values of Pr.

**Figure 5:**
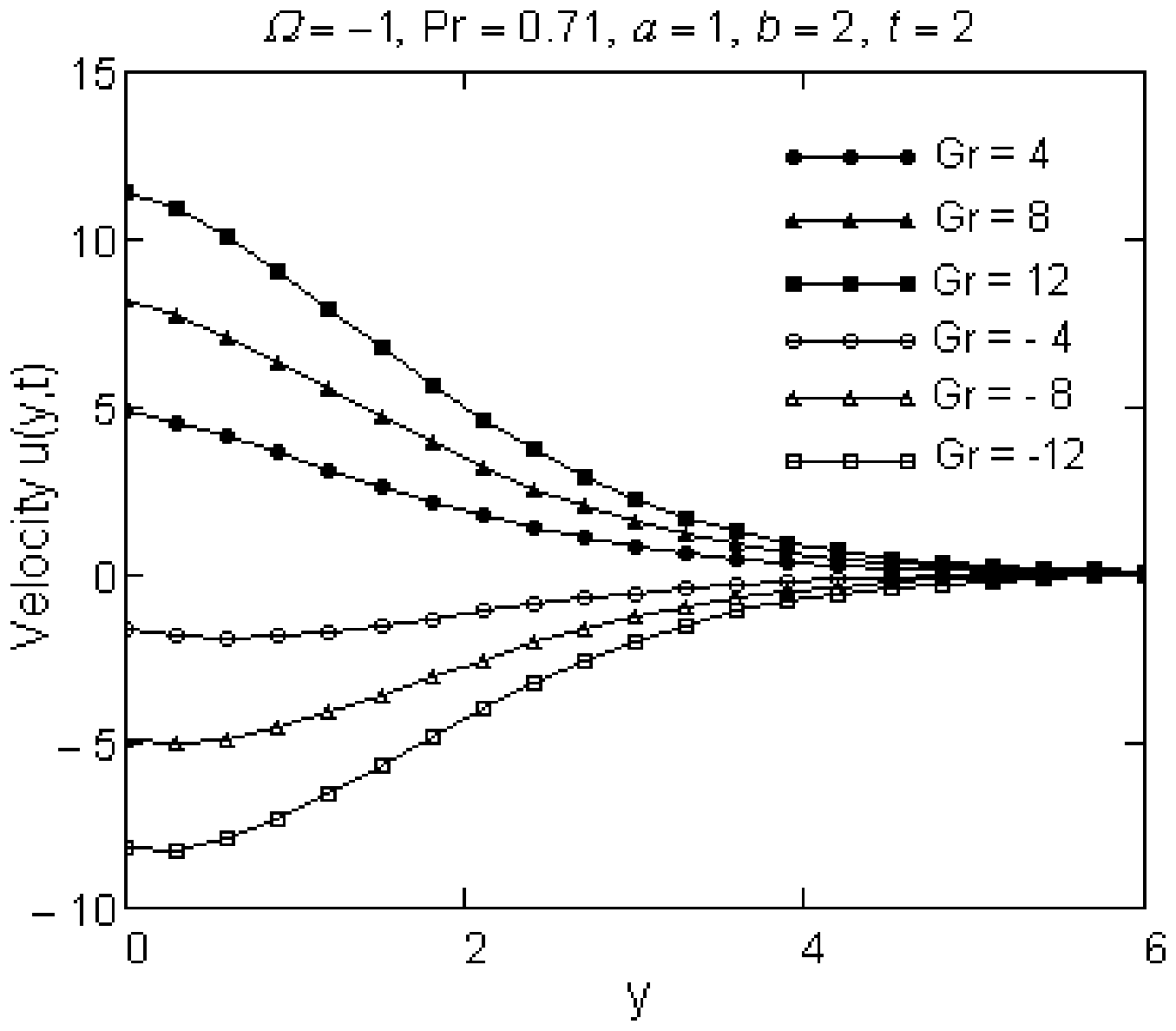
Velocity profiles for different values of Gr.

**Figure 6:**
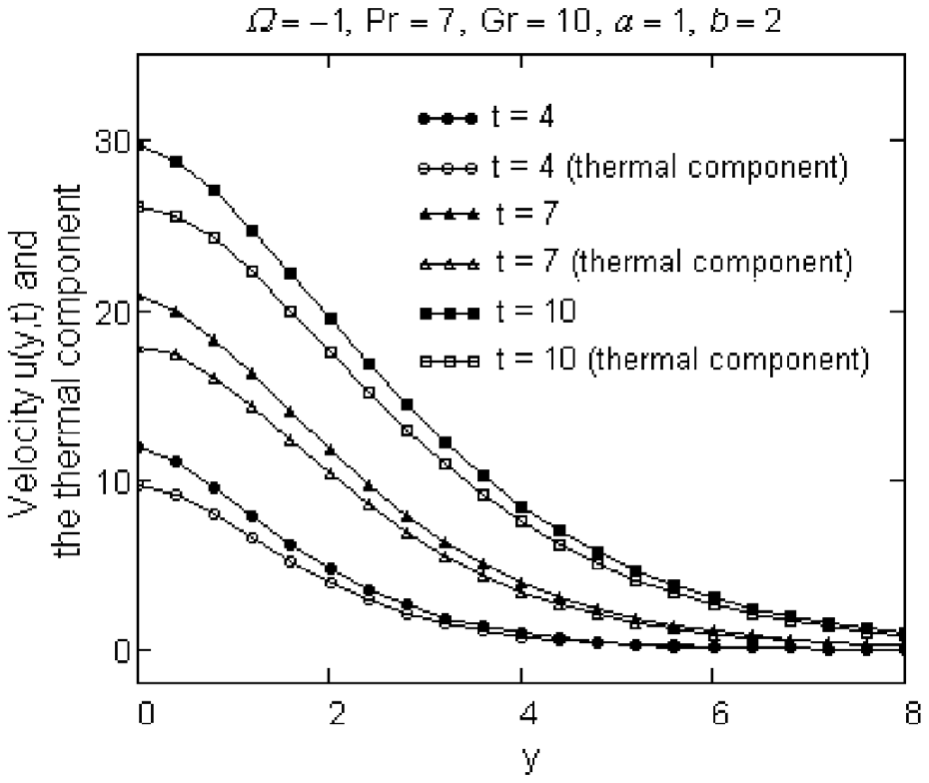
Comparison between the velocity 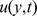 and its thermal component 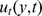 for different values of *t*.

The effects of Pr and Gr numbers on the fluid motion are presented in Figs. 4 and 5. Especially, they are significant in the vicinity of the plate and the fluid velocity smoothly decreases from maximum values at the wall to the zero value for increasing y. The velocity of the fluid, as it results from Fig. 4, is a decreasing function with respect to Pr. This is consistent with the fact that the fluids with high Prandtl number have greater viscosity and move slowly. Velocity profiles against y are also presented for different positive and negative values of Gr in Fig. 5. Physically, 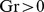 corresponds to an externally cooled plate [8] and 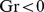 corresponds to an externally heated plate. It is clearly seen that the velocity is an increasing function with respect to Gr in case of the cooling of the plate. This is because an increase in Gr leads to an increase in the buoyancy force which causes the increase in the fluid velocity. But a reverse effect is found in case of the heating of the plate. For comparison purposes, profiles of velocity 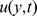 and of its thermal component 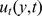 against *y* are shown in Fig. 6 for different values of *t*. It is clearly seen that the free convection effects are significant and the boundary layer thickness increases in both cases.

**Figure 7:**
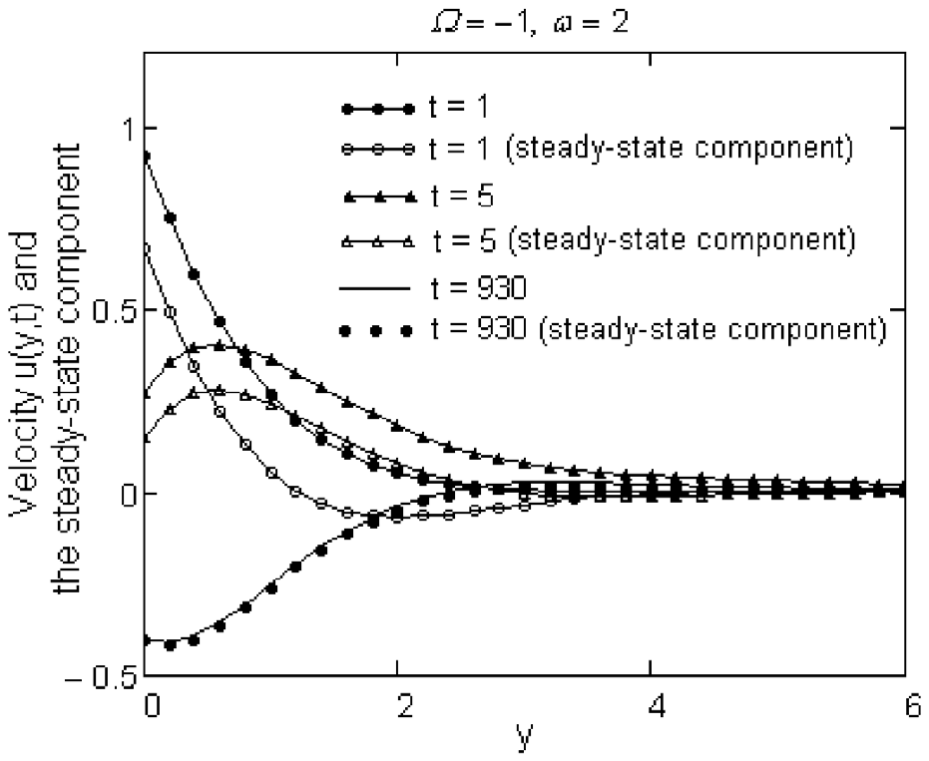
The required time to reach the steady-state for sine oscillations of the boundary shear.

**Figure 8:**
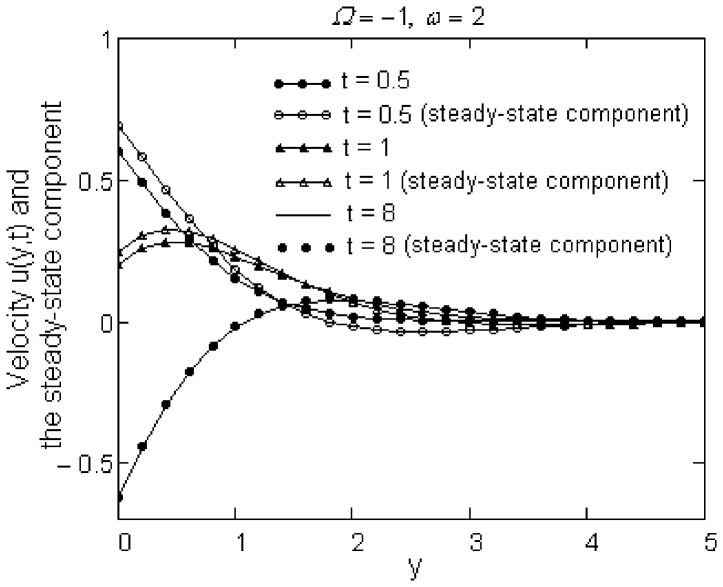
The required time to reach the steady-state for cosine oscillations of the boundary shear.

Finally, the required time to reach the steady-state for motions due to a plate that applies oscillatory shear stresses to the fluid is determined by Figs. 7 and 8. At small values of time, the difference between starting and steady-state solutions is rather large. This difference, which is more significant for the sine oscillations of the shear, rapidly decreases for cosine oscillations of the shear on the boundary and the required time to reach the steady-state in this case (namely, 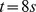) is much smaller in comparison with that corresponding to sine oscillations of the boundary shear stress. Of course, this time also depends on the constants 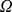 and 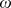, but we did not include the corresponding graphics here.

## Conclusions

The purpose of this work is to provide exact solutions for the unsteady natural convection flow of an incompressible viscous fluid near an infinite plate with shear stress boundary conditions and exponential heating. More exactly, after time 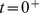, the exponentially heated plate applies an arbitrary shear stress to the fluid. The governing coupled linear partial differential equations are solved using Laplace transforms and closed-form solutions are obtained for velocity and temperature without any restriction. As it results from (2), the radiative effects are not taken into consideration in our work. However, in view of the reference [19] (see also the work of Magyari and Pantokratoras [26]), it is clearly seen that the solutions of the same problem with radiative effects are immediately obtained from our solutions by substituting Pr by 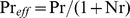 where Nr is the radiation-conduction parameter. Consequently, the study of a natural convection flow with or without radiative effects is practically the same problem. The temperature of the fluid, as well as its velocity, is the same for an infinite set of values of parameters Pr and Nr which correspond to the same effective Prandtl number 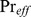.

Finally, in order to emphasize the influence of pertinent parameters on the temperature, Nusselt number and velocity distributions, and to get some physical insight of the obtained results, two special cases are considered and some numerical calculations and graphs have been carried out. The main findings are:

Exact solutions for temperature and velocity are obtained in terms of the complementary error function. They satisfy all imposed initial and boundary conditions.
The velocity of the fluid 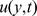 can be written as a sum of its mechanical and thermal components 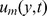, respectively 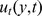.
The thermal boundary layer, as well as the temperature of the fluid, increases in time and decreases with respect to the Prandtl number Pr.
Nusselt number profiles decrease with respect to Pr near the surface of the plate and then increase. Nusselt number values tend to a steady value as the time increases.
The effects of Pr and Gr numbers on the fluid motion are significant and fluids with higher Prandtl number or smaller positive Grashof number move slowly.
The fluid velocity, as expected, is an increasing function with respect to Gr in the case of the cooling of the plate. A reverse effect is observed in case of the heating of the plate.
The required time to reach the steady-state is much smaller in the case of cosine oscillations in comparison to sine oscillations of the shear stress on the boundary. This is obvious, because at time 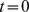 the shear stress on the boundary is zero for the sine oscillations of the shear.
